# Exploiting Sphingo- and Glycerophospholipid Impairment to Select Effective Drugs and Biomarkers for CMT1A

**DOI:** 10.3389/fneur.2020.00903

**Published:** 2020-08-25

**Authors:** Davide Visigalli, Giovanna Capodivento, Abdul Basit, Roberto Fernández, Zeeshan Hamid, Barbora Pencová, Chiara Gemelli, Daniela Marubbi, Cecilia Pastorino, Adrienne M. Luoma, Christian Riekel, Daniel A. Kirschner, Angelo Schenone, José A. Fernández, Andrea Armirotti, Lucilla Nobbio

**Affiliations:** ^1^DINOGMI, University of Genoa, Genoa, Italy; ^2^IRCCS Ospedale Policlinico S. Martino, UO Clinica Neurologica, Genoa, Italy; ^3^Analytical Chemistry Lab, Fondazione Istituto Italiano di Tecnologia, Genoa, Italy; ^4^Department of Physical Chemistry, Faculty of Science and Technology, University of the Basque Country, Leioa, Spain; ^5^DIMES, University of Genoa, Genoa, Italy; ^6^IRCCS Ospedale Policlinico S. Martino, UO Oncologia Cellulare Genoa, Genoa, Italy; ^7^Department of Biology, Boston College, Boston, MA, United States; ^8^The European Synchrotron, ESRF, Grenoble, France

**Keywords:** CMT1A, biomarker, drug, myelin, lipid metabolism, Schwann cell, demyelination, peripheral neuropathy

## Abstract

In Charcot–Marie–Tooth type 1A (CMT1A), Schwann cells exhibit a preponderant transcriptional deficiency of genes involved in lipid biosynthesis. This perturbed lipid metabolism affects the peripheral nerve physiology and the structure of peripheral myelin. Nevertheless, the identification and functional characterization of the lipid species mainly responsible for CMT1A myelin impairment currently lack. This is critical in the pathogenesis of the neuropathy since lipids are many and complex molecules which play essential roles in the cell, including the structural components of cellular membranes, cell signaling, and membrane trafficking. Moreover, lipids themselves are able to modify gene transcription, thereby affecting the genotype–phenotype correlation of well-defined inherited diseases, including CMT1A. Here we report for the first time a comprehensive lipid profiling in experimental and human CMT1A, demonstrating a previously unknown specific alteration of sphingolipid (SP) and glycerophospholipid (GP) metabolism. Notably, SP, and GP changes even emerge in biological fluids of CMT1A rat and human patients, implying a systemic metabolic dysfunction for these specific lipid classes. Actually, SP and GP are not merely reduced; their expression is instead aberrant, contributing to the ultrastructural abnormalities that we detailed by X-ray diffraction in rat and human internode myelin. The modulation of SP and GP pathways in myelinating dorsal root ganglia cultures clearly sustains this issue. In fact, just selected molecules interacting with these pathways are able to modify the altered geometric parameters of CMT1A myelinated fibers. Overall, we propose to exploit the present SP and GP metabolism impairment to select effective drugs and validate a set of reliable biomarkers, which remain a challenge in CMT1A neuropathy.

## Introduction

Charcot–Marie–Tooth (CMT) neuropathies are a group of inherited diseases affecting peripheral nerve function. Among them, CMT type 1A (CMT1A) is the most prevalent type, displaying characteristic dys/demyelination that still rules its own classification (1, 2). The genetic defect responsible for CMT1A, a 1.5-Mb duplication on chromosome 17p11.2 containing the *PMP22* gene, was identified more than 20 years ago (3–6); however, this revelation has not led to the ultimate comprehension of CMT1A genotype–phenotype correlation yet nor to an effective therapy (7, 8). In fact, CMT1A patients sharing the same genetic defect experience a remarkable phenotypic diversity and severity (9–11).

As for other hereditary neuromuscular diseases in which gene therapy proved to be effective, the negative regulation of *PMP22* expression represents the ideal biological endpoint and therapeutic goal (12–14). To date, in contrast to preclinical studies, clinical trials in CMT1A patients aimed to down-regulate *PMP22* showed negative results (7, 15–17). Furthermore, gene therapy, in addition to the obvious technical difficulties, seems to be still poorly acceptable for CMT1A patients in which life expectancy and quality of life are not dramatically affected (18, 19). Alternative therapeutic options should be explored, as previously suggested (20–25).

In this context, we focused on dys/demyelination as a critical target downstream of the *PMP22* defect, assuming that the refined knowledge of lipid metabolism is essential to effectively address this aspect (26).

Indeed myelin chemical composition, structure, and physiology are intimately related and altered in several dysmyelinating neuropathies, including CMT1A (27–32). Moreover, peripheral myelin distress is caused by mutations in a variety of genes, including those coding for enzymes involved in lipid biosynthesis and metabolism (33–37). Notably, several molecules modulate the activity of these enzymes and are used as real drugs in pathological conditions (38). Recent studies demonstrated that soy phospholipid and high-fat neutral lipid-enriched diets improve myelination in CMT1A and CMT1E animal models (30, 39). It has also been shown that changes in the circulating lipid profile may be related to the onset, activity, and progression of some important human diseases, including myelin disorders (40–42).

In spite of these observations, lipid metabolism still remains largely under-explored in CMT neuropathies, including CMT1A (39). In fact, it is vital to identify and characterize lipid species and their changes, biological role, and mutual interaction (43).

In the present study, we addressed this gap by a multi-disciplinary and technologically advanced approach, performing a comprehensive study of lipid metabolism both in experimental and in human CMT1A.

We found that sphingolipid and glycerophospholipid (SP and GP) pathways are mainly responsible for the perturbed lipid metabolism already described in CMT1A (30). We also found a systemic alteration of SP and GP metabolism in experimental and in human CMT1A biological fluids. Notably, the specific targeting of just these pathways was able to improve the altered structural parameters of CMT1A myelinated fibers *in vitro*.

## Materials and Methods

### Animal Model

The CMT rat, an animal model of CMT1A neuropathy originally developed in K-A Nave laboratory, was used for the experiments (44). Sixty-day-old heterozygous CMT1A animals and wild-type (WT) littermates of both sexes were used for most of the experiments unless otherwise specified. We confirm that all methods were performed in accordance with relevant guidelines/regulations. In particular, the research protocols presented in this study are conducted in accordance with the ARRIVE guidelines and are included in those reviewed and approved by the OPBA (Institutional Animal Welfare Body) and by the Italian Ministry of Health (project number approval 798/2016-PR).

### Cell Culture and Drug Administration

Myelinating dorsal root ganglia (DRG) cultures were established from 15-day-old embryos as previously described (29, 45–47). Briefly, 35–40 DRG were removed from each embryo, incubated for 30 min with 0.25% trypsin in Hanks' solution, and minced to obtain a suspension of DRG cells. The cells were washed and dissolved in complete medium made up of neurobasal medium (Invitrogen) supplemented with 15% newborn calf serum, ascorbic acid (100 μg/ml), and nerve growth factor at 5 ng/ml. This suspension was plated on collagen-coated ACLAR dishes at a density of 15 × 10^4^ cells/dish.

Both CMT1A and WT DRG cultures were grown in complete medium and chronically treated with different molecules interfering with SP and GP metabolism (see also **Figure 3A**). In particular, the cultures were treated every other day for 30 days with oleyl-L-α-lysophosphatidic acid (LPA) (Na^+^ salt) (Sigma-Aldrich, L7260), L-α-phosphatidic acid (PA) (Na^+^ salt) (Sigma-Aldrich, P9511), CDP-choline (Na^+^ salt) (Sigma-Aldrich, C0256), VO-OHpic trihydrate (a PTEN inhibitor) (Sigma-Aldrich, V8639), phosphatidylinositol tris-3,4,5-phosphate (Na^+^ salt) (PIP3) (Matreya, Restek Superchrom, 1775-1), desipramine hydrochloride (Sigma-Aldrich, D3900), sphingomyelin (Sigma-Aldrich, S0074), L-serine (Sigma-Aldrich, S4311), and 2-hydroxy oleic acid (2OHOA) (Sigma-Aldrich, SML0256). The vehicle was represented by distilled water (ddH_2_O) for L-serine, PIP3, desipramine, and CDP-choline, Dulbecco's phosphate-buffered saline (DPBS) (Gibco, 14190-144) for LPA, PA, and dimethyl sulfoxide (Sigma, D8418) for VO-OHpic, and 2OHOA and absolute ethanol for sphingomyelin, respectively. At the end of each treatment, the DRG cultures were processed for advanced quantitative neuropathology (see below).

Preliminary dose–response experiments were performed for each molecule, including the vehicle to define safety and effectiveness (data available at request). The sample size of the treatment groups was calculated with PASS 11 (http://www.ncss.com/software/pass/). The power analysis was performed in *a priori* manner. Type I error was given with 5% and type II error was given with ≥90%. Myelin density and internode length served as main outcome measures for the power analysis. Assuming a complete recovery of the phenotype as the endpoint, mean post-treatment differences of 5 × 10^−5^ units for myelin density and of 13.67 units for internode length are required for the CMT1A DRG cultures. We calculated a sample size of three cultures for each group to reach a statistical power higher than 90% (97 and 98%, respectively). The most effective dosage for the molecules displaying a significant effect on the geometric parameters of myelinated fibers was validated on a second round of experiments. In particular, we analyzed the effect of LPA (0.1 μM), PIP3 (2 μM), L-serine (0.5 mM), and VO-OHpic (0.1 μM) on CMT1A and WT DRG cultures.

### Samples From Human Subjects

The subjects involved in this exploratory study included 15 healthy controls (eight females and seven males, mean age: 47 ± 11 years, range: 27–67 years) and 28 patients with clinical and genetic diagnosis of CMT1A (18 females and 10 males, mean age: 55 ± 15 years, range: 22–82 years). Our small cohort of patients displayed a mean CMTNS of 13.35 ± 5.78 (95% CI, 10.64–16.06) in agreement with the most frequent phenotype observed in CMT1A populations (48). We confirm that all methods were performed in accordance with relevant guidelines/regulations. The Regional Ethics Committee (CER Liguria) approved the current study protocol (project number approval 545REG2015), and the patients gave written informed consent according to the Helsinki Declaration as revised in 2013. Peripheral blood (5 ml) from CMT1A patients and controls was added to collection tubes containing a clot activator and spun to obtain the serum fraction. Serum was immediately frozen and stored at −80°C in 500-μl aliquots to perform lipidomics; repeated freezing and thawing was avoided. The archived sural nerve biopsies from patients with CMT1A and from subjects who underwent nerve biopsy for suspected peripheral neuropathy were also used to perform X-ray diffraction analysis.

### Isolation of PNS Myelin

Rat sciatic nerves were used to prepare myelin-enriched fraction as previously described (32, 49).

### Rat CSF and Serum Collection

Cerebrospinal fluid (CSF) collection was performed according to Liu et al. (50). Briefly, the rats were anesthetized with an intraperitoneal injection of ketamine/xylazine cocktail (100:10 mg/kg) and tightly fixed on ear bars to immobilize the head of the animals. The skin at the base of the neck was removed and the CSF was collected through a cisterna magna puncture technique, and immediately frozen at −80°C. Serum collection was performed from the retro-orbital plexus by a capillary tube. The blood was clotted at room temperature and centrifuged prior to freezing the serum at −80°C. All the procedures were performed according to the ARRIVE guidelines.

### Lipidomics by LC–MS/MS

Lipids were extracted from sciatic nerves, purified myelin, and biological fluids by using the Bligh–Dyer protocol (51). In brief, 2 ml of 1:2 chloroform/methanol mixture (v/v) was added to the vials and vortexed for 30 s. Chloroform (0.5 ml) and water (0.5 ml) were then sequentially added and thoroughly mixed after each addition. The samples were then centrifuged for 15 min at 3,500 × g at room temperature (RT). At the end of the process, the organic (lower) phase (~1.5 ml) was transferred to glass vials. The aqueous phases were re-extracted to increase the overall recovery. The organic phases from both extractions were pooled in a glass vial and dried under a nitrogen stream. The extracted lipids were re-dissolved in 0.1 ml of a 9:1 methanol/chloroform solution and analyzed by liquid chromatography coupled to high-resolution mass spectrometry (MS).

Untargeted lipidomics of lipid extracts was performed on a UPLC Acquity system coupled to a Synapt G2 QToF high-resolution mass spectrometer. The lipids were separated on a CSH C18 column (1.7 M particle size, 2.1 × 100 mm). Mobile phase A consisted of acetonitrile/water (60:40) with 10 mM ammonium formate, and mobile phase B consisted of acetonitrile/isopropyl alcohol (10:90) with 10 mM ammonium formate. The following gradient program was applied: 15% B for 1 min after injection, then increased to 60% B in 9 min, then to 75% B in 8 min, and then to 100% B for a further 2.5 min. An isocratic 100% B step was then maintained for 2.5 min, and the column was subsequently reconditioned to 15% B for 2 min. Total run time was 25 min with the following conditions: flow rate−0.4 ml/min, column temperature−55°C, and injection volume−6 μl. The instrument was operated in positive electrospray ionization (ESI) mode. The MS source parameters were as follows: capillary and cone voltages were set at 2.8 kV and 30 V, respectively, source and desolvation temperature were set at 100 and 450°C, respectively, and desolvation gas and cone gas (N_2_) flows were set at 800 and 50 L/h, respectively. The mass spectra were recorded in MSe mode, with MS/MS fragmentation performed in the trap region on the instrument. Low-energy scans were acquired at a fixed 4 eV collision energy, and high-energy scans were acquired using a collision energy ramp from 20 to 40 eV. The spectra were recorded at a mass resolution of 20,000 in the range of 50–1,200 m/z. The scan rate was set to 0.3 spectra per second. A leucine–enkephalin solution (2 ng/ml) was continuously infused in the ESI source (4 μl/min) and acquired every 30 s for real-time mass axis recalibration (52). All the samples were run in random order. Quality control samples, consisting of a pool of all the samples, were acquired and were used to assess system suitability, performance, and reproducibility.

### LC-MS/MS Data Analysis

Raw data were analyzed using MarkerLynx software (Waters Inc.) to re-align the observed peak and extract all the relevant features (53). The feature list was then analyzed with MetaboAnalyst 4.0, a web-based metabolomic data processing tool (54). Following principal component analysis (PCA), heatmap analyses were performed to detect the features showing significant changes between the groups. Lipid identification was then performed by interrogating the web-based algorithm HMDB using the accurate mass measured for each feature. The following adduct species were searched: [M + H]+, [M + NH_4_]+, [M + H–H_2_O]+, [M + Na]+, [M + H−2H_2_O]+, [M + CH_3_CN + H]+, [M + NH_4_-H_2_O]+, [M + isopropanol + Na]+, [M + 2CH_3_CN + H]+, [M + 2Na–H]+, and [M + K]+. A maximum of 5 ppm tolerance in mass accuracy was allowed. Whenever possible, feature annotation was also supported by tandem mass data (MSe mode) and class-specific retention time (55). Based on the putative ID of the statistically relevant features, pathway analysis was performed using a hypergeometric test for overrepresentation and using a *Rattus norvegicus* or *Homo sapiens* metabolite background.

### X-ray Diffraction Analysis

#### Rat Sciatic Nerve

The fixed dissected sciatic nerves, prepared from rats that were 1.5 and 3 months old, were tied off at their ends and sealed, in contact with excess buffered fixative, in 0.7-mm-diameter quartz capillary tubes. X-ray diffraction spectra were recorded from the nerves using nickel-filtered, single-mirror-focused CuKα radiation from a fine-line source on a 3.0-kW Rigaku X-ray generator that was outfitted with a linear, position-sensitive detector, as described in Avila et al. (56). The myelin periodicity was calculated from the positions of the Bragg reflections in the patterns. The relative amount of multilamellar myelin was determined by measuring the total integrated intensity of the Bragg reflections (*M*) above background (*B*). The fraction of scattered intensity that is due to myelin is then *M*/(*M* + *B*) (56).

#### Human Sural Nerve Biobsy

Small-angle X-ray scattering (SAXS) experiments were performed at the ID13 beamline of the European Synchrotron Radiation Facility (ESRF). The pink beam from an undulator was monochromated to a wavelength of λ = 0.09755 nm, with Δλ/λ~2 × 10^−4^ by a liquid-N2-cooled Si crystal. Epon-embedded longitudinal and transversal sections of control human and CMT1A sural nerve samples were deposited on plain mesh TEM grids (57). A protein crystallography microgoniometer with on-axis optical microscopy allowed obtaining SAXS patterns at specific locations and extracting bilayer periods from the observed lamellar orders (58).

The X-ray beam was focused by Kirkpatrick–Baez double mirrors and collimated by an aperture to a 5 × 5 μm^2^ full-width half-maximum spot at the sample position (59). Longitudinal and transversal sections of Epon-embedded samples were probed in transmission geometry at specific positions using a protein microcrystallography goniometer (58). The sample-to-detector distance was calibrated by an Ag-behenate standard to be 485.3 mm. SAXS patterns were recorded by a MarCCD detector (Rayonix) with typical exposure times of 60 s/pattern. The patterns were displayed and analyzed using the FIT2D software.

### Advanced Neuropathology in DRG Cultures

Following pharmacological manipulation of SP and GP metabolism, DRG cultures were carefully rinsed in DPBS (Gibco, 14190-144), fixed for 20 min in 4% paraformaldehyde (Sigma-Aldrich, P6148) in DPBS, rinsed again in DPBS, and incubated for 30 min into a permeabilizing/blocking buffer (GSDB buffer) containing 33.3% normal goat serum (DAKO, X0907), 10% Triton-100 (Sigma-Aldrich, T8787), phosphate buffer (240 mM), pH 7.4, and sodium chloride (4 M). Incubation with the anti-myelin basic protein antibody, aa 129–138 clone 1 (Sigma-Aldrich, MAB 382, 1:300), was performed at 4°C in GSDB buffer overnight. After careful rinsing in DPBS, the cells were incubated with Alexa Fluor 594 goat anti-mouse IgG (H+L) secondary antibody (Invitrogen, A11005) at 1:400 dilutions for 1.5 h at 25°C. The cell nuclei were stained with 4′,6-diamidino-2-phenylindole dihydrochloride; fluoropure grade (300 nM) (Invitrogen, D1306) and ACLAR dishes were sealed on slides.

Images from the whole DRG culture (80 images/culture and an average number of quantified myelinated internodes of 1 × 10^4^) were acquired, using the ×20 objective, with an Olympus PROVIS AX60 microscope connected to an Olympus DP70 digital camera. A morphometric evaluation of the digitized images was performed using an *ad hoc* Image Pro-Plus macro that we recently established in our laboratory, using the Image Pro-Plus Software (Immagini e Computer, Rho, Milan, Italy) (see Figure S10 and Video S1). This semi-automated tool allows a detailed morphometric analysis of the critical parameters for myelinated fibers physiology including (i) myelinated area (i.e., number of myelinated pixels/total pixels on the micrograph) and (ii) internode length and its relative frequency functions. Actually, this allowed us to reliably distinguish CMT1A myelinated internode from the control one for all the parameters analyzed (see Figure S10).

### Statistical Analysis

The results are presented as mean ± SEM unless otherwise specified. Outliers, whenever present, were identified and removed through the ROUT method (*Q* = 1%). For myelinated area and internode length, we performed the D'Agostino–Pearson normality test to assess the type of data distribution. Therefore, statistical differences were determined using the non-parametric Mann–Whitney test. For internode length frequency distributions, we performed the non-parametric Kruskal–Wallis test followed by Dunn's multiple-comparisons test. Metabolomics statistics, including PCA, pathway analysis, and volcano plot for the heatmap data, were all performed with MetaboAnalyst software. Statistical differences were considered to be significant when *P* < 0.05. Unless otherwise specified, statistical analysis was performed using the Graph Pad 7.0 (Prism) software.

## Results

### SP and GP Pathways Are Mainly Responsible for Lipid Alteration in CMT1A Myelin

To gain a complete overview of CMT1A peripheral nerve lipid composition, we performed a comprehensive lipidomic analysis both on lipid fraction extracted from sciatic nerve homogenates and on purified myelin.

**Figure 1:**
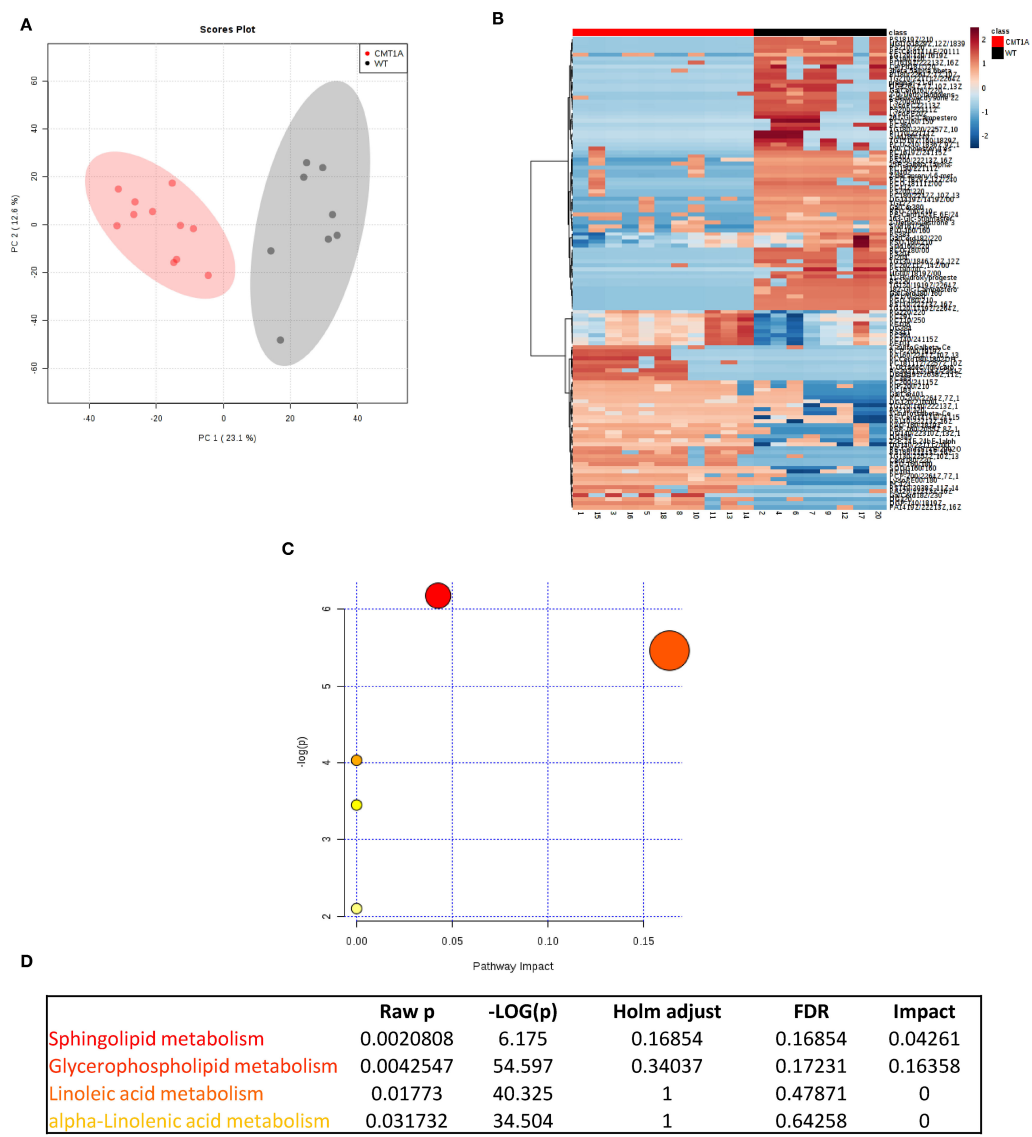
Multivariate data analysis (MVA) of rat sciatic nerve lipidome clearly discriminates CMT1A from controls displaying a specific impairment of sphingolipid and glycerophospholipid metabolism. **(A)** Score plot from principal component analysis (PCA) of untargeted lipidomics data. PCA was able to reliably discriminate CMT1A nerves (red, *n* = 11) from the wild type ones (black, *n* = 8). **(B)** Heatmap generated with the most significant features (with highest fold change and statistical significance) detected by MVA. One hundred twenty-one features were mainly responsible for the difference between the two groups. **(C)** Graph presenting the probability (y-axis) and the impact (x-axis) that a pathway is responsible for the difference shown in lipidomic profiles. Each circle represents a specific lipid pathway; the circle size represents the number of hits per pathway. Red–orange–yellow–white diminishing scale represents the degree of involvement in lipidomic profiles. **(D)** Table indicating only the significant pathways presented in the graph.

We found that the CMT1A nerve homogenate displays a distinctive lipid profile compared to that of WT littermates (Figure 1A). In particular, we identified 121 lipid species that significantly differ between the two conditions. Among them, SP and GP mostly contributed to this remarkable diversity (Figures 1B–D). Notably, the SP and GP species in CMT1A were not merely reduced as it could be expected by the impaired ability of Schwann cells (SCs) to mount the whole lipid biosynthetic transcriptional program during myelination (30); instead we found an incorrect pattern of SP and GP characterized by the presence of lipid species where these were highly increased alongside lipid species where these were extremely decreased (Figures S1, S2 and Table S1).

To map this aberrant lipid profile directly into the intact rat sciatic nerve, we performed MALDI-IMS [Figure S3; (60)]: the clustering algorithm classified the two genotypes in clearly separate groups just in the endoneurium. Importantly, we also confirmed SP and GP as the most compromised pathways (Figures S4–S8).

**Figure 2:**
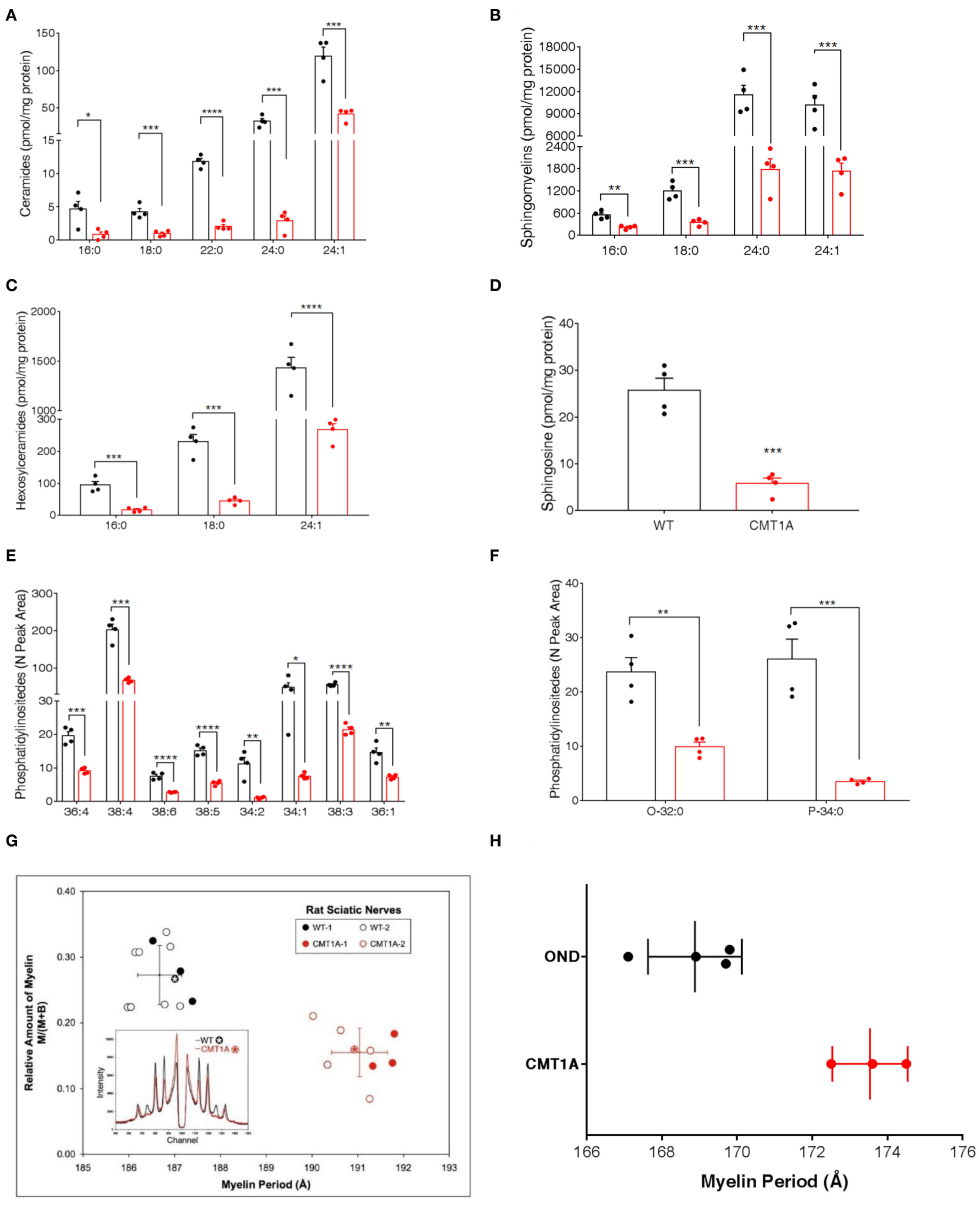
CMT1A myelin displayed perturbed sphingolipid (SP) and glycerophospholipid composition and ultrastructural abnormalities. The most abundant SP species were analyzed by an optimized protocol of targeted mass spectrometry. This method uses 25 standards to calculate the absolute concentration (nM) of sphingolipids of interest. **(A–D)** Ceramides, sphingomyelins, hexosylceramides, and sphingosine were all reduced in the CMT1A samples. **(E,F)** Phosphatidylinositides were the lipid species mainly altered in CMT1A myelin by shotgun untargeted analysis. The compound names are presented on the y-axis, while different acyl chains, sature, or insature, are presented on the x-axis. Data are presented as mean ± mean of standard error. Wild type (WT) (black), CMT1A (red) *n* = 4. Statistics was calculated with unpaired *t*-test, two-tailed. ****p < 0.0001, ***p < 0.001, **p < 0.01, and *p < 0.05. **(G)** X-ray diffraction performed on WT and CMT1A rat sciatic nerves from four different litters at 1.5 months (filled symbols) and at 3 months (open symbols) of age. The whole nerves were fixed in 2.5% glutaraldehyde in cacodylate buffer, pH 7.4, for 24 h. In the graph, the relative amount of myelin vs. myelin period is reported. The CMT1A rats showed a significantly reduced myelin content and an enlarged myelin periodicity compared to the WT littermates, which demonstrates the presence of myelin ultrastructural alterations in this neuropathy (WT black, *n* = 12; CMT1A red, *n* = 9, unpaired *t*-test, two-tailed; *p* < 0.0001 for both relative amount of myelin and myelin periodicity). The inset shows examples of the diffraction patterns, expressed as diffracted intensity vs. detector channel number. The patterns correspond to the data points marked by the asterisks. The small shift in the positions of the Bragg peaks indicates differences in periodicity, and the weaker peaks indicate less myelin. The middle region of each pattern, approximate channel numbers 950–1,050, is central scatter from the direct beam around the beam stop and, therefore, is excluded from the analysis. **(H)** X-ray diffraction performed on human sural nerve biopsies of patients affected by CMT1A compared to patients affected by other neurological diseases (OND). The nerves were fixed in buffered glutaraldehyde, processed for electron microscopy, and embedded in Epon, which accounts for the differences in periodicities with the results in **(G)**. Notably, the CMT1A patients (red, *n* = 3) displayed enlarged myelin periodicity compared to the control patients (OND, black, *n* = 4, unpaired *t*-test, two-tailed; *p* < 0.01) as was found in the CMT1A rat. Data are presented as mean ± standard deviation.

To unambiguously demonstrate that, among lipids, SP and GP mainly contributed to CMT1A myelin pathology, we performed targeted lipidomics on CMT1A myelin-enriched fraction. Similar to the sciatic nerve, the SP and the GP pathways were deeply altered (Figures 2A–F).

Indeed this aberrant lipid composition might also account for the ultrastructural changes of the CMT1A myelin membrane that have been described by us and other groups (29, 30). Thus, to strengthen the issue, we used, for the first time, X-ray diffraction that can precisely detect subtle changes in myelin periodicity to assess both internode myelin quantity and quality in sciatic and sural nerves from CMT1A rats and human patients, respectively (Figures 2G,H). Notably, we found a significantly reduced amount of myelin and a substantial widening of myelin period, which are consistent with the aberrant lipid composition [Figures 2G,H; (30, 61, 62)].

### Modulation of SP and GP Pathways Affects CMT1A Myelin Physical Structure

To demonstrate a link between SP and GP imbalance and aberrant myelin physical structure in CMT1A, we took advantage from DRG cultures an *in vitro* model of the disease originally developed in our laboratory (29, 45, 63).

**Figure 3:**
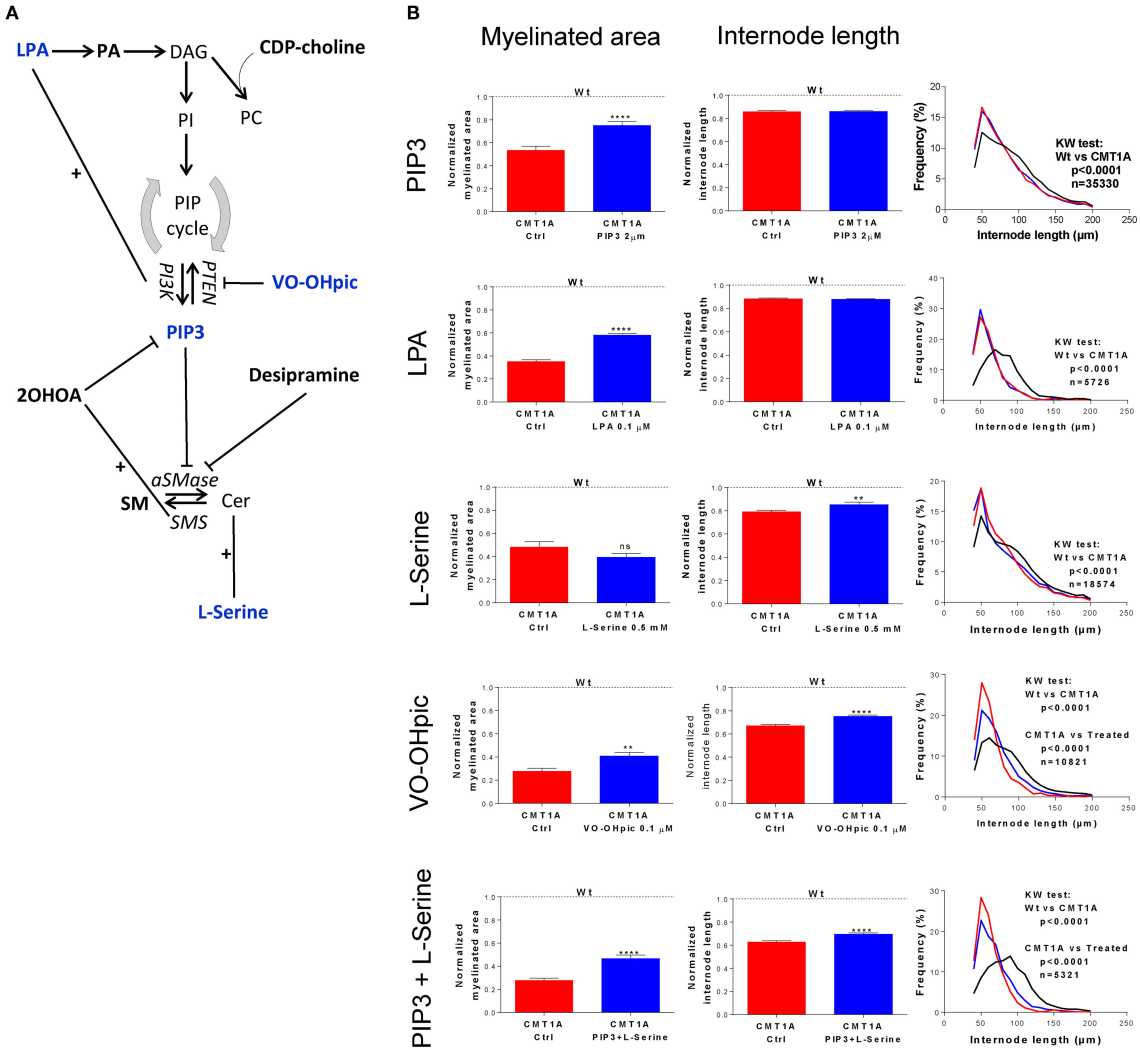
Modulation of sphingolipid (SP) and glycerophospholipid (GP) pathways affects the CMT1A myelin physical structure. **(A)** Schematic illustration of treatment schedule adopted to demonstrate the specific involvement of SP and GP in CMT1A myelinopathy. The CMT1A and WT DRG cultures were chronically treated with several molecules selected for their proven efficacy on SP and GP pathway modulation in the presence of 15% newborn calf serum, ascorbic acid (100 μg/ml final concentration), and nerve growth factor at 5 ng/ml final concentration. In particular, we analyzed the effects on myelination of LPA, PA, CDP-choline, PIP3, VO-OHpic, 2OHOA, desipramine, SM, and L-serine. **(B)** Advanced neuropathology (see also the Video S1) performed on dorsal root ganglia (DRG) myelinated fibers allowed us to select PIP3, LPA, VO-OHpic, and L-serine as the most effective molecules. Interestingly, we found that these molecules improved the CMT1A myelinopathy—a reduced amount of myelinated fibers and shortening of the internode length—in a different way. In fact, while PIP3 and LPA significantly increased the amount of myelinated fibers without any effect on their structure [CMT1A Ctrl (*n* = 180) vs. CMT1A PIP3 (*n* = 215), mean ± SD: 0.53 ± 0.43 vs. 0.75 ± 0.50; CMT1A Ctrl (*n* = 405) vs. CMT1A LPA (*n* = 511), mean ± SD: 0.35 ± 0.27 vs. 0.58 ± 0.40), L-serine and VO-OHpic just increased the internode length (CMT1A Ctrl (*n* = 102) vs. CMT1A L-serine (*n* = 113), mean ± SD: 0.79 ± 0.12 vs. 0.85 ± 0.23; CMT1A Ctrl (*n* = 128) vs. CMT1A VO-OHpic (*n* = 155), mean ± SD: 0.67 ± 0.11 vs. 0.75 ± 0.11], suggesting the existence of at least two independent mechanisms essential for correct myelination. This hypothesis is further strengthened by the cumulative effect of PIP3 and L-serine simultaneous administration to CMT1A DRG cultures, which was able to improve both the quantity and the quality of pathological myelin [CMT1A Ctrl (*n* = 112) vs. CMT1A PIP3+L-serine (*n* = 145), myelinated area mean ± SD: 0.27 ± 0.20 vs. 0.46 ± 0.36; CMT1A Ctrl (*n* =112) vs. CMT1A PIP3+L-serine (*n* = 145), internode length mean ± SD: 0.62 ± 0.10 vs. 0.69 ± 0.12]. LPA, lysophosphatidic acid; PA, phosphatidic acid; DAG, diacylglycerol; CDP-choline, cytidine-5′-diphospho-choline; PI, phosphoinositide; PC, phosphatidylcholine; PIP, phosphatidylinositol phosphate; PTEN, phosphatase and tensin homolog; VO-OHpic, a PTEN inhibitor; PI3K, phosphatidylinositol-3-kinase; PIP3, phosphatidylinositol tris-3,4,5-phosphate; 2OHOA, 2-hydroxy oleic acid; SM, sphingomyelin; aSMase, acid sphingomyelinase; SMS, sphingomyelin synthase; Cer, ceramide. For myelinated area and internode length, we performed the D'Agostino–Pearson normality test to assess the type of data distribution. Therefore, statistical differences were determined using the non-parametric Mann–Whitney test (*n* represents the number of analyzed images in at least three biological replicates). For internode length frequency distributions, we performed non-parametric Kruskal–Wallis test followed by Dunn's multiple-comparisons test (*n* represents the total number of analyzed internodes). ns, not significant; **p < 0.01 and ****p < 0.0001.

In particular, we chronically treated CMT1A and WT DRG cultures with different molecules interfering with both SP and GP metabolism (Figure 3A).

We found that four drugs were effective in improving the geometric parameters of CMT1A myelinated fibers (Figure 3B). In particular, PtdIns(3,4,5)P3 (PIP3) and lysophosphatidic acid (LPA) were able to significantly increase just the amount of myelinated area without any effect on the internode length (PIP3: 41.5%, *p* < 0.0001; LPA: 65.7%, *p* < 0.0001); conversely, L-serine significantly improved only the internode length (7.5%, *p* < 0.01). Notably, VO-OHpic, a quite specific inhibitor of PTEN, a phospholipid phosphatase that we found to be significantly increased in CMT1A (Figure S10), was able at the same time to positively affect both of these aspects (myelinated area: 48.1%, *p* < 0.01; internode length: 11.9%, *p* < 0.0001). Actually, L-serine and VO-OHpic affected the internode frequency distribution, shifting the function back to the WT condition (Figure 3B). Interestingly, when we performed a combined treatment with L-serine and PIP3, we observed an improvement of both internode parameters (myelinated area: 70.3%, *p* < 0.0001; internode length: 11.2%, *p* < 0.0001) following the VO-OHpic treatment, suggesting the existence of at least two independent molecular mechanisms that affect the CMT1A myelin assembly. Notably, these effects did not occur in the WT condition, reinforcing the specificity of CMT1A SP and GP pathway impairment (Figure 3B).

### SP and GP Pathways Are a Proper Source of CMT1A Blood Biomarkers

**Figure 4:**
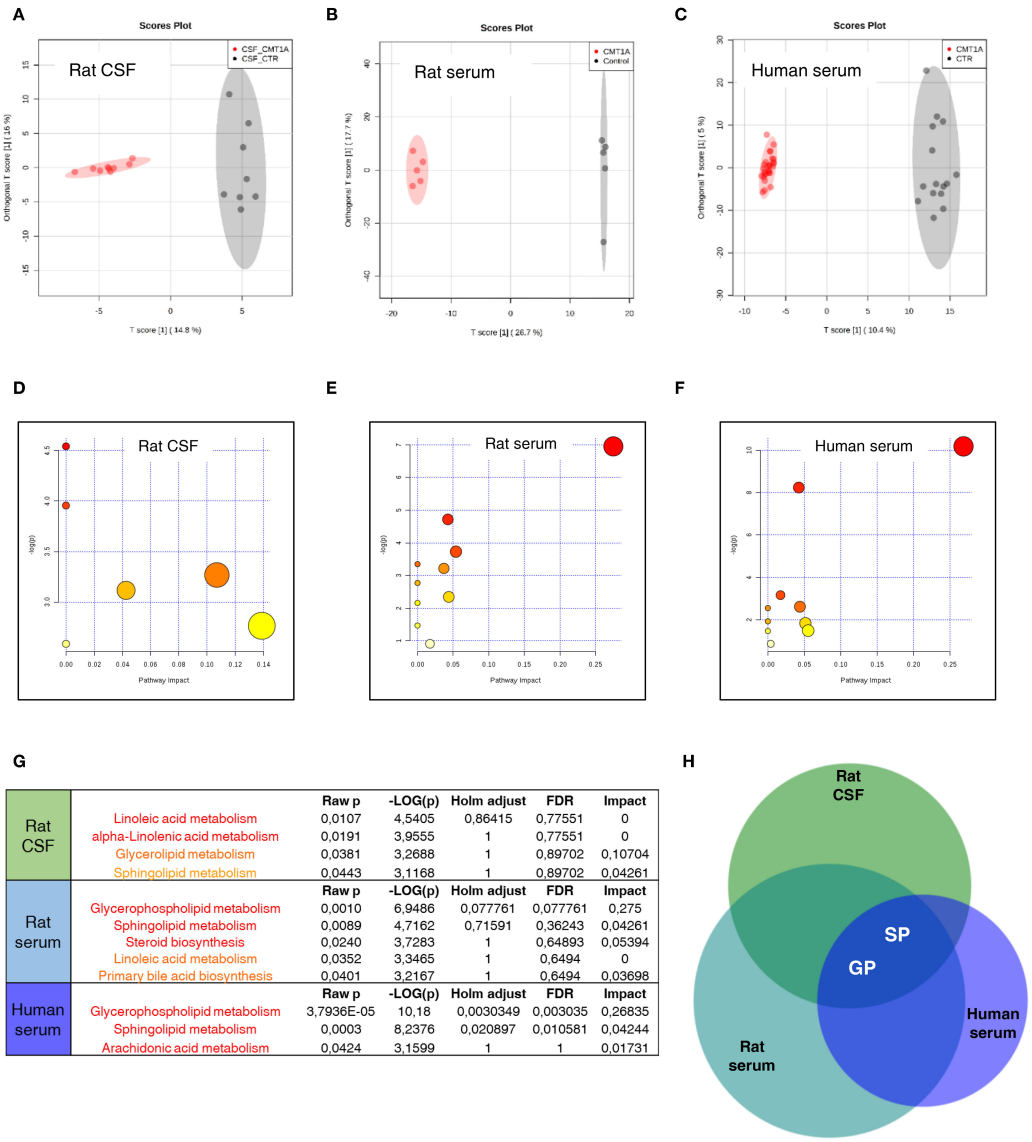
Sphingolipid (SP) and glycerophospholipid (GP) pathways present a reliable source of CMT1A wet biomarkers. **(A)** Score plot from Orthogonal Projections to Latent Structures Discriminant Analysis (OPLS-DA) of untargeted lipidomics data. **(A)** OPLS-DA analysis was able to reliably discriminate CMT1A CSF (red, *n* = 9) from the wild type (WT) one (black, *n* = 8). **(B)** Corresponding OPLS-DA analysis of rat serum lipidome displayed a clear separation between the two phenotypes (WT, black, *n* = 5 and CMT1A, red, *n* = 5). **(C)** Untargeted lipidomics was also performed on the serum of 15 healthy donors and 28 CMT1A patients. Notably, OPLS-DA analysis demonstrated a clear clustering of the subjects into two groups according to the genotype. **(D–F)** Graphs presenting the probability (y-axis) and the impact (x-axis) that a pathway is responsible for the differences shown in lipidomic profiles. Each circle represents a specific lipid pathway; the circle size represents the number of hits per pathway. Red–orange–yellow–white diminishing scale represents the degree of involvement in lipidomic profiles. **(G)** Table indicating only significant pathways presented in the graphs. **(H)** A comparative pathway analysis (Venn diagram) shows that the dysregulation of SP and GP metabolism is mainly responsible for the difference between CMT1A and controls in both rat biofluids and the serum of human subjects. The diagram was designed by Biovenn online software (http://www.biovenn.nl/).

To test whether the massive change of SP and GP metabolism that we found in CMT1A myelin might be traced into the circulatory system, we performed a complete lipid profile in CSF and serum from CMT1A rat and human patients. We identified a unique and specific lipid profile in the CSF of CMT1A rats compared to controls (Figure 4A). In particular, we recognized 37 features that clearly discriminate the two experimental groups, as shown in the corresponding heatmap (Figure S11 and Table S1). Also, in the rat serum, the CMT1A lipid profile strongly diverged from that of the WT littermates, supporting the notion that a systemic impairment of lipid metabolism is present in this neuropathy (Figure 4B). Notably, serum lipidomics in the rat revealed that 67 features, mostly belonging to SP and GP metabolism, especially contributed to the unique lipid profile of CMT1A (Figure S11 and Table S1). Owing to the highly consistent results obtained in experimental CMT1A, we extended our study to human subjects by performing lipidomics on the serum collected from 28 genetically defined CMT1A patients and 15 age-matched healthy donors, without any evident comorbidity with diseases that compromise lipid metabolism. As reported in the score plot, the CMT1A patients significantly clustered from healthy controls (Figure 4C). In particular, 141 features were dysregulated between the two groups. Once again, the majority of these features belong to SP and GP pathways (Figure S11 and Table S1). Interestingly, among them, we found remarkable alterations in the levels of known myelin-enriched lipids, including sulfatides and gangliosides, supporting the notion that the CMT1A lipid perturbation present in peripheral myelin may be tracked in the blood. Of note is the fact that these changes were definitely huge: most of the identified features increased or decreased by more than hundreds of thousands of times in human serum, an optimal condition to perform a future biomarker discovery activity. Indeed the comparative pathway analysis between CMT1A rat and human biological fluids showed that the alteration of SP and GP metabolism was the common feature (Figures 4D–H).

## Discussion

Myelin lipid deficiency and its partial rescue in response to a lipid-enriched diet have been recently shown in the CMT1A rat model (30).

The present study provides evidence for the first time that, following a comprehensive analysis of the whole lipidome, just SP and GP are responsible for the CMT1A perturbed lipid metabolism in the nerve, myelin, and biological fluids.

These results definitely extend and better define earlier works (30, 39). Of note also in our study is that different SP and GP species (including phosphatidylcholines) are altered in CMT1A, but with species increased as well as decreased, complicating the issue. In this context, it is well-known that the unique lipid stoichiometry of myelin directly influences its ultrastructure and physiology, thereby regulating nerve conduction (26, 31). In fact, myelin is subjected to different physical and functional rearrangements during development, including progressive thickening, lengthening along fiber internodes, and increasing of conduction velocity. All these features are altered in CMT1A and never reach normal values (64–68). Indeed changes in CMT1A myelin geometric parameters have already been shown by us and other groups (29, 30, 64, 69). Moreover, here we demonstrate by X-ray diffraction analysis a flawed physical structure of internode myelin in both experimental and human CMT1A.

To link SP and GP imbalance with CMT1A myelin architecture, we investigated the morphometric changes of the myelinated internode in DRG cultures treated with different molecules affecting just these lipid pathways (32, 70–79). Of note is that most of the SP and GP species displaying a greater change do not only have a structural role in myelin but are also able to impact on signaling and transcription itself (34).

Among all the tested molecules, we found that L-serine, PIP3, LPA, and VO-OHpic were able to improve CMT1A myelin internode, in contrast to an earlier work in which lipid supplement was unable to influence these parameters (30). Moreover, the effect of our treatments was specific for the CMT1A condition as we did not observe any effect in wild-type cultures.

Indeed these molecules deeply interfere with normal and pathological myelination. In fact, L-serine is a key polar amino acid required for SP synthesis, being an essential substrate for SPTLC1, the rate-limiting enzyme of SP metabolism; peripheral myelin is particularly sensitive to SP composition and change (80, 81). PIP3, in glia, triggers autonomous cell wrapping during myelination, and an imbalance of PIP homeostasis is at the basis of altered longitudinal myelin growth and myelin outfolding formation (37, 73, 82, 83). LPA is an early precursor of PI that regulates crucial signaling in myelin, including embryonic SC migration, myelination, and cell-to-axon segregation; PI also prevents SC apoptosis through activation of PI3K, a key enzyme for PIP3 homeostasis (70). Finally, VO-OHpic is a synthetic specific inhibitor of PTEN, an enzyme that works in concert with Dlg1 as myelination inhibitor, and deletion of PTEN in adult SCs is able to reactivate myelin growth (77, 84, 85).

We would like to highlight that these molecules act on the PI3K/PTEN pathway, a well-known CMT1A hub, downstream to NRG1, further sustaining the importance of SP and GP metabolism in myelin development and maintenance.

We found that these molecules affect myelinated area and internode length in different ways: in fact, while PIP3 and LPA significantly increased the amount of myelinated fibers without any effect on their structure, L-serine and VO-OHpic just increased the internode length, suggesting the existence of at least two independent mechanisms essential for correct myelination. This hypothesis is further strengthened by the cumulative effect of PIP3 and L-serine simultaneous administration, which was able to improve both the quantity and the quality of the myelin internode.

Trying to further explain our results, we envisage that these treatments are able to impact on SC differentiation and proliferation, two biological processes highly compromised in CMT1A (86, 87). Indeed LPA and PIP3, improving the myelinated area through the PI3K/PTEN pathway, may directly trigger the CMT1A SC differentiation program (70, 73). The positive effect of L-serine and VO-OHpic on CMT1A internode length might instead be due to their ability to inhibit proliferation. In fact, the relation between increased proliferation and shortening of internode is well-known (88–90). Interestingly, several studies demonstrate that SPTLC1 and PTEN, sensitive to L-serine, and VO-OHpic, respectively, are also able to modulate proliferation independently from their normal enzymatic activity (91–93). Further studies are needed to demonstrate the correlation between abnormal cellular proliferation and myelin structure impairment in CMT1A.

Overall, our results display a leading role of SP and GP metabolism in CMT1A impaired myelination.

Notably, the same SP and GP dysregulation emerges from the lipidomics performed on biological fluids both in experimental and in human CMT1A. Our results strongly recommend SP and GP as a promising source of disease biomarkers. Actually, while there is no need for CMT1A diagnostic biomarkers, great effort is underway to identify disease severity biomarkers to stage patients, to follow disease progression, and to monitor drug efficacy in clinical trials (94). To date, transcriptional biomarkers in skin biopsies of experimental and human CMT1A have been proposed, demonstrating that disease severity can be related to cutaneous mRNA expression (14, 95). Moreover, a study performed on the plasma of CMT1A patients highlighted an increase of protein catabolism and the mobilization of membrane lipids involved in inflammatory signaling as further potential sources of biomarkers (96). Nevertheless, we are still far from having reliable and clinically acceptable disease severity biomarkers. Owing to our analysis of the CMT1A serum lipid profile, a most reliable fluid in clinical practice, it is our opinion that the present study represents a critical step toward this direction.

Finally, despite that our study is focused on CMT1A, it is intriguing that defects of lipid metabolism have been described in several other peripheral neuropathies. Among them, there are not only inherited dysmyelinating CMT—including CMT1B, CMT4B1, and CMT4J—but also HSAN type 1 and diabetic neuropathy (37, 97–100). Even some axonal CMT2 neuropathies display a dysregulation of lipid metabolism, which further supports a critical role of bioactive lipid species in peripheral nerve physiology (42, 101).

## Conclusions

The lipidome profiling in experimental and in human CMT1A reported herein for the first time and the established previously unknown alterations in SP and GP metabolism expand the spectrum of molecular changes in CMT1A. The consistent systemic altered lipidome of affected patients deeply supports the use of lipid serum biomarkers in CMT1A and possibly other CMT neuropathies in which lipid biosynthesis and myelin remodeling are compromised. These novel data provide insights into the pathological alterations in the CMT1A neuropathy at a molecular level and could potentially contribute to the development of novel disease-modifying approaches.

## Data Availability Statement

All datasets generated for this study are included in the article/Supplementary Material. Raw data are available from the corresponding author on reasonable request.

## Ethics Statement

The studies involving human participants were reviewed and approved by the Regional Ethics Committee (CER Liguria) (project number approval 545REG2015). The patients/participants provided their written informed consent to participate in this study. The animal study was reviewed and approved by the OPBA (Institutional Animal Welfare Body) and the Italian Ministry of Health (project number approval 798/2016-PR).

## Author Contributions

DV and GC planned the experiments and analyzed the data. DV wrote the manuscript. GC performed immunohistochemistry on DRG cultures and supervised the serum collection from human patients. DM and CP prepared the samples for the analyses from animal material. AB and ZH acquired targeted and untargeted lipidomic data. BP did sample preparation and the MALDI-IMS experimental work. RF performed the MALDI-IMS data analysis and lipid identification. CG enrolled patients and collected the serum samples and clinical data. JF coordinated the MALDI-IMS experiments and took part in the interpretation. CR performed the X-ray scattering experiments at the ESRF and analyzed the data. DK and AL undertook the small-angle X-ray diffraction on aldehyde-fixed nerves and analyzed the data. DK contributed to the writing of the manuscript. AS contributed to the manuscript discussion. AA contributed to the LC-MS/MS experimental design and data analysis. LN designed and supervised the study, performed and analyzed the experiments on DRG cultures, and wrote the manuscript. All authors contributed to the article and approved the submitted version.

## Conflict of Interest

The authors declare that the research was conducted in the absence of any commercial or financial relationships that could be construed as a potential conflict of interest.
