# Supplementary material for: Exploiting Sphingo- and Glycerophospholipid Impairment to Select Effective Drugs and Biomarkers for CMT1A
Source: Front Neurol. 2020 Aug 25;11:903. doi: 10.3389/fneur.2020.00903 (PMC7477391; doi:10.3389/fneur.2020.00903)
Supplement: Supplementary file 2 [file Data_Sheet_1.PDF]

Supporting Information for

Exploiting sphingo- and glycerophospholipid impairment to select effective drugs and biomarkers for CMT1A

Davide Visigalli, Giovanna Capodivento, Abdul Basit, Roberto Fernández, Zeeshan Hamid, Barbora Pencová, Chiara Gemelli, Daniela Marubbi, Cecilia Pastorino, Adrienne M. Luoma, Christian Riekel, Daniel A. Kirschner, Angelo Schenone, José A. Fernández, Andrea Armirotti, Lucilla Nobbio

Correspondence to: Lucilla Nobbio; email: lnobbio@neurologia.unige.it

This PDF file includes:

Supplementary text
S1-S11 Figs.
S1 Table

Methods

Lipidomics by MALDI-IMS

We performed MALDI-IMS (matrix-assisted laser desorption/ionization imaging mass spectrometry) to determine the lipid composition of rat sciatic nerve and their relative abundance in the three main anatomic areas of this nerve.
Briefly, 15 µm thick sections from 12 animals (6 CMT1A and 6 WT) were explored in the experiments. DAN (1,5-diaminonaphtalene) and MBT (2-mercaptobenzotiazole) were used for negative- and positive-ion mode respectively [59, 60]. Lipid assignment in the images was based on the comparison between the experimental m/z ratio (mass-to-charge) and the species in the software's database (<33,000 lipid species plus adducts), the LIPID MAPS database (www.lipidmaps.org) and those identified by LC-MS/MS [61]. Mass resolution was set to 60,000 at m/z = 400 Da in a 550-1200 Da mass window. The spectra in each experiment were grouped using k-means and attending to the similarity of the lipid fingerprint of each pixel. Changes in lipid composition between CMT1A and WT nerves were assessed using ANOVA. Hematoxylin and eosin (H&E) stain was used to take optical images of a consecutive section to compare with the IMS images and to verify that the lipid distribution follows the histology of the sample.
Detailed description of the protocol used in IMS experiments may be found elsewhere [62].

SMase activity assay

To assess aSMase activity we adjusted an available assay to our purpose (Zhang et al., 2001). In particular, rat sciatic nerve homogenates were added to individual wells of a 96-well microtiter plate containing acidic buffer (50 mM sodium acetate, pH 5.0). A defined amount of SM (0.5 mM) was added to each well and the plate was incubated for 3h at 37°C. Then, an enzymatic cocktail, consisting of 8 U/ml of alkaline phosphatase, 0.2 U/ml of choline oxidase, 2 U/ml of horseradish peroxidase, and 2 U/ml of AR in 100 µl of reaction buffer, was added and the plate was incubated for further 30 minutes at 37°C in the dark. For each sample, the relative negative control obtained by SM subtraction was also analysed. Following the reaction, the microtiter plate was read using a fluorescence microplate reader with excitation and emission wavelength at 560 and 587 nm, respectively. A standard curve was prepared by making serial dilutions (from 0.1953 µU to 100 µU) from a standard stock solution of *Bacillus cereus* SMase. For each sample SMase activity was calculated from the fluorescence difference between the sample and its relative negative control. Resulting values were interpolated with the standard curve to obtain absolute SMase activity units (mU).

Real-Time PCR

RT-qPCR was conducted on a LightCycler 480 System using the Probes Master Kit as already described (Visigalli et al., 2016). Primers and probes were designed using the Universal Probe Library Assay Design Center for RT-qPCR (S1 Table).

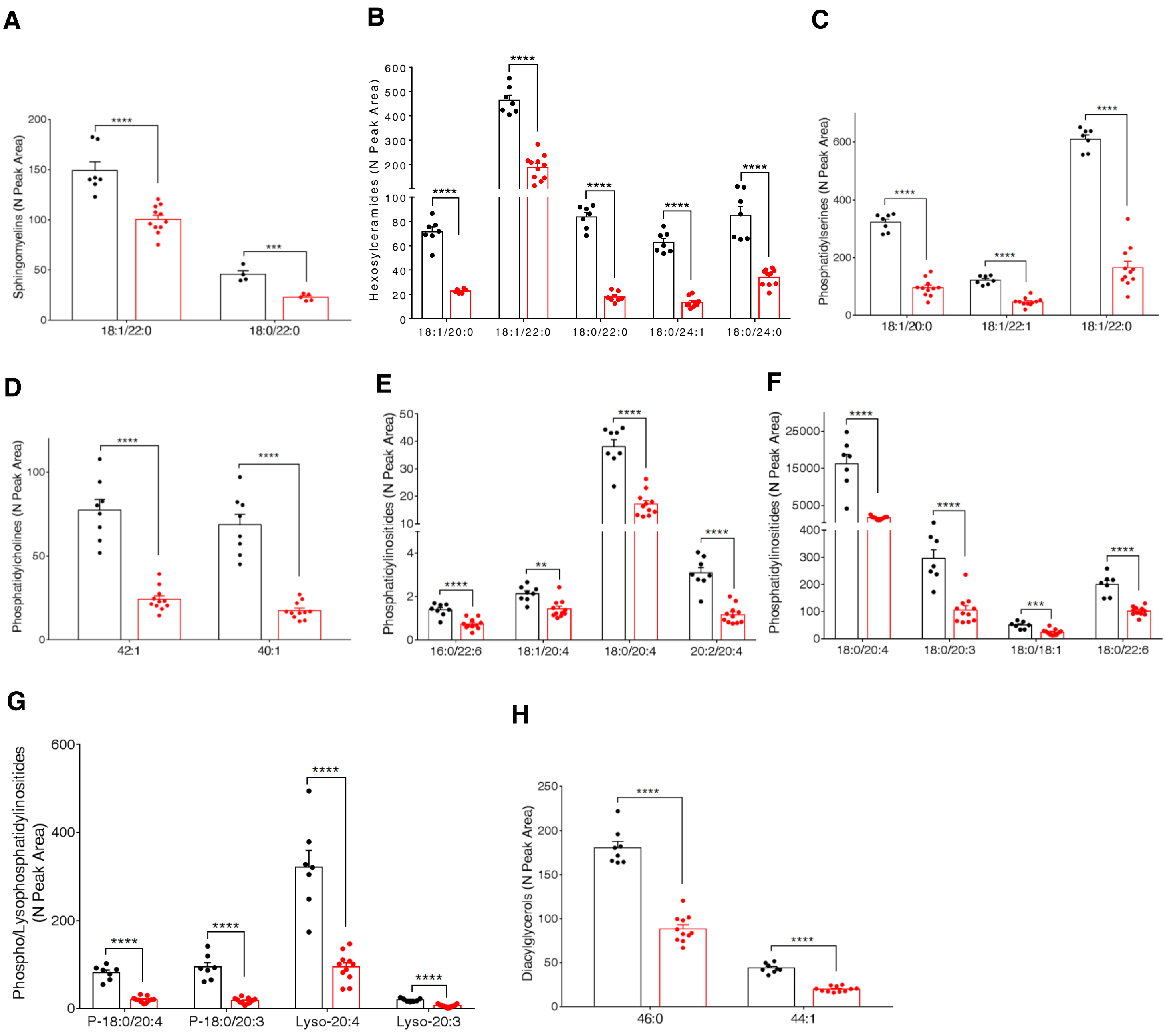

S1 Fig. Lipid species decreased in CMT1A rat sciatic nerves.

(A, B) Sphingolipid species (sphingomyelins and hexosylceramides) mainly decreased in CMT1A (red) nerves. **(C-H)** Phospholipid species (phosphatidylserines, phosphatidylcholines, phosphatidylinositides and diacylglycerols) mainly decreased in CMT1A. Compound names were represented on y-axis, while different acyl chains, sature or insature, were represented on the x-axis. Data were represented as mean of peak area ± mean of standard error (SEM). WT (black) $N = 8$, CMT1A (red) $N = 11$. Statistics was calculated with unpaired t test, two-tailed. **** = $p < 0.0001$, *** = $p < 0.001$, ** = $p < 0.01$.

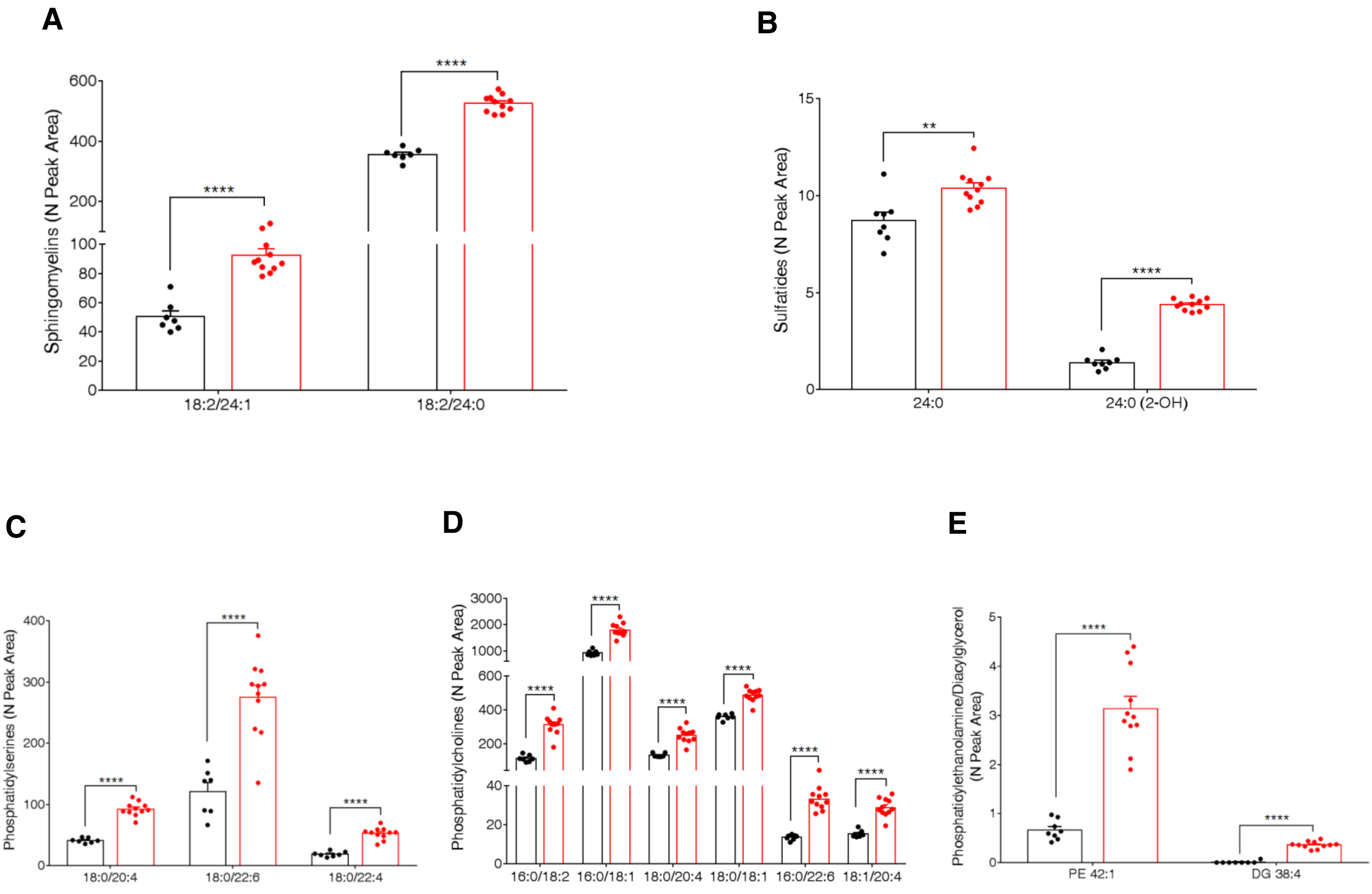

S2 Fig. Lipid species increased in CMT1A rat sciatic nerves.

(A, B) Sphingolipid species (sphingomyelins and sulfatides) mainly increased in CMT1A (red) nerves. **(C-E)** Phospholipid species (phosphatidylserines, phosphatidylcholines, phosphatidylethanolamines and diacylglycerols) mainly increased in CMT1A. Compound names were represented on y-axis, while different acyl chains, sature or insature, were represented on the x-axis. Data were represented as mean of peak area ± mean of standard error (SEM). WT (black) $N = 8$, CMT1A (red) $N = 11$. Statistics was calculated with unpaired t test, two-tailed. **** = $p < 0.0001$, ** = $p < 0.01$.

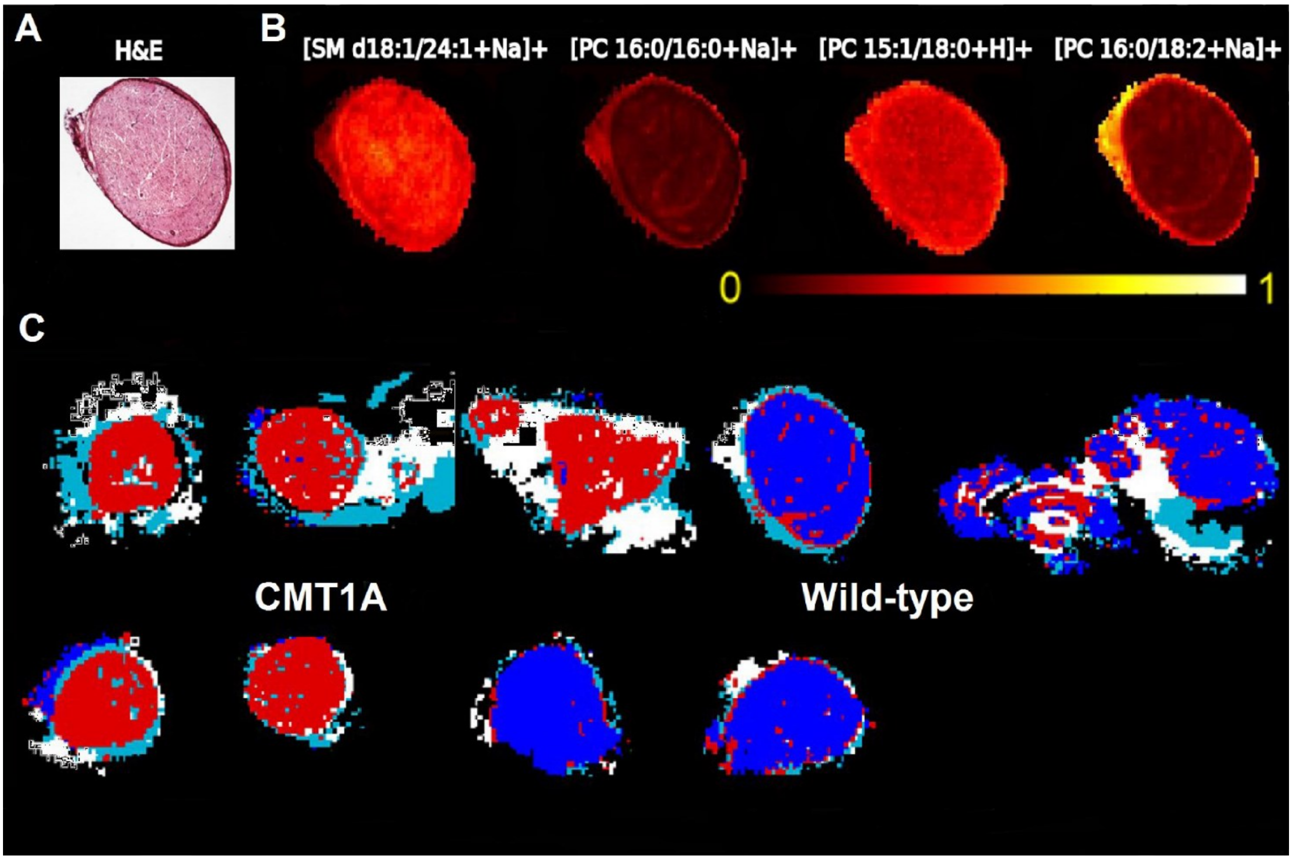

S3 Fig. MALDI-IMS of rat sciatic nerve confirms the unique lipid profile of CMT1A compared to controls.

(A, B) Representative images from an IMS experiment to map the distribution of lipids in rat sciatic nerves. **(A)** Histological sections are used for comparison with the lipid species assigned to each region of the same sample by IMS. **(B)** During the experiment, the whole surface of the nerve section is examined to collect information about the composition and distribution of the analyzed lipids at every point (m/z = 550/1200 Da). Each peak in the mass spectrum corresponds to a unique lipid species for which specific localization and amount are obtained and represented using a black-red-orange-yellow-white scale. Examples of different lipid species are reported in **(B)**. **(C)** K-means analysis of nine experiments of MALDI-IMS, setting the number of segments to four. The differences in lipid composition result in a clear separation between CMT1A (red) and Wild-type (dark blue) fibres. Conversely, the adipose tissue of both WT and CMT1A appear as two segments (white and light blue, respectively) spread along the nine experiments, pointing to a not-significant lipid difference between the two groups. All experiments recorded at 25 mm/pixel in positive-ion mode. Abbreviations: SM = Sphingomyelin; PC = Phosphatidylcholine

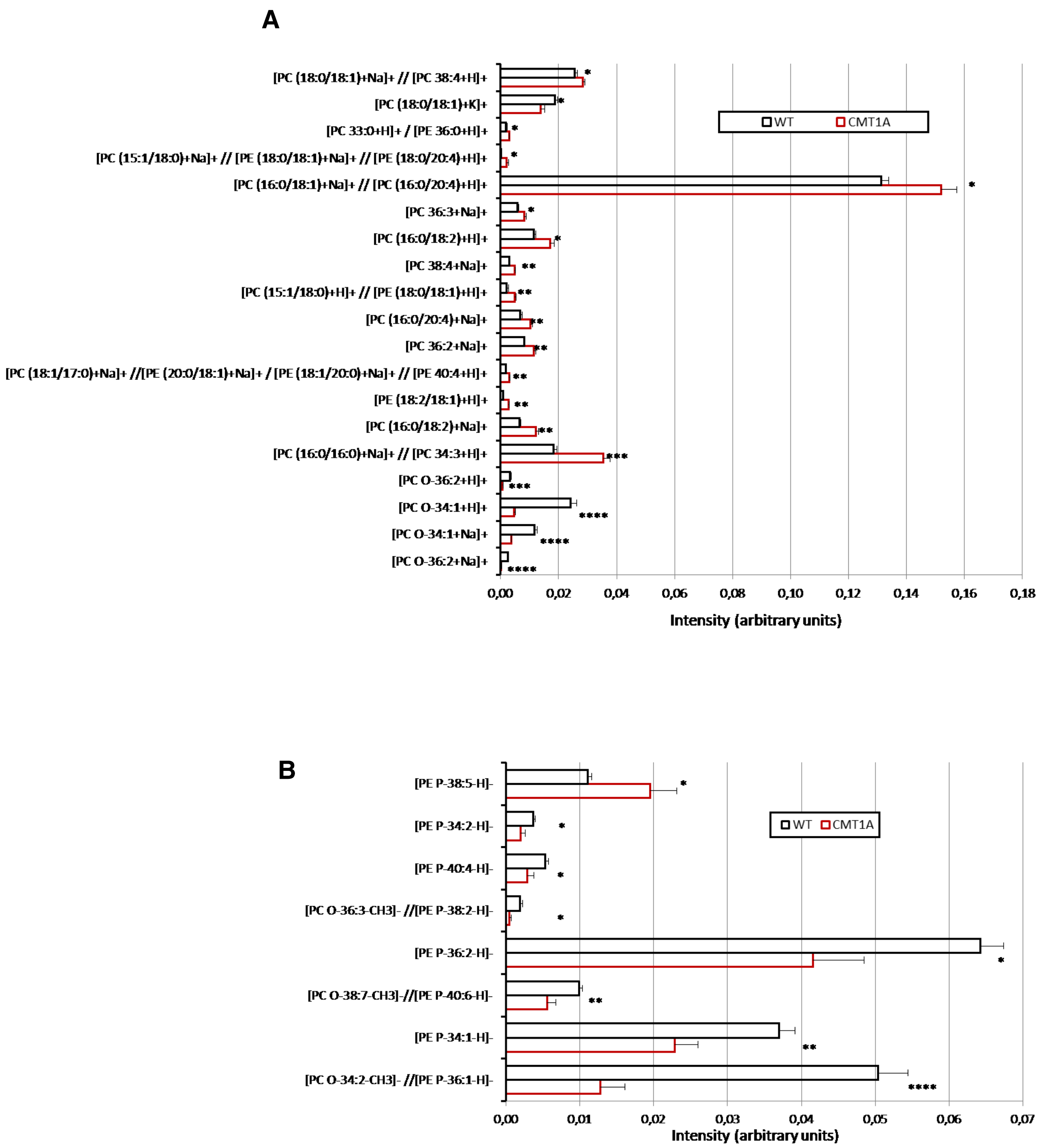

S4 Fig. MALDI-IMS on CMT1A and WT rat sciatic nerve sections.

PC & PE (including plasmalogens) detected in positive- **(A)** and negative-ion mode **(B)** that experience a significant change between WT and CMT1A sciatic nerve endoneurium. Most of the m/z may be assigned to (or contain contribution from) more than one species. The lipids are organized in order of significance. PC were assumed to be ethers and PC vinyl-ethers, as it has been demonstrated by LC-MS/MS analysis. Diacyl species are slightly more abundant in CMT1A while ether/vinyl ether species are significantly more abundant in CMT1A. Please, note that the results in both polarity modes are consistent.

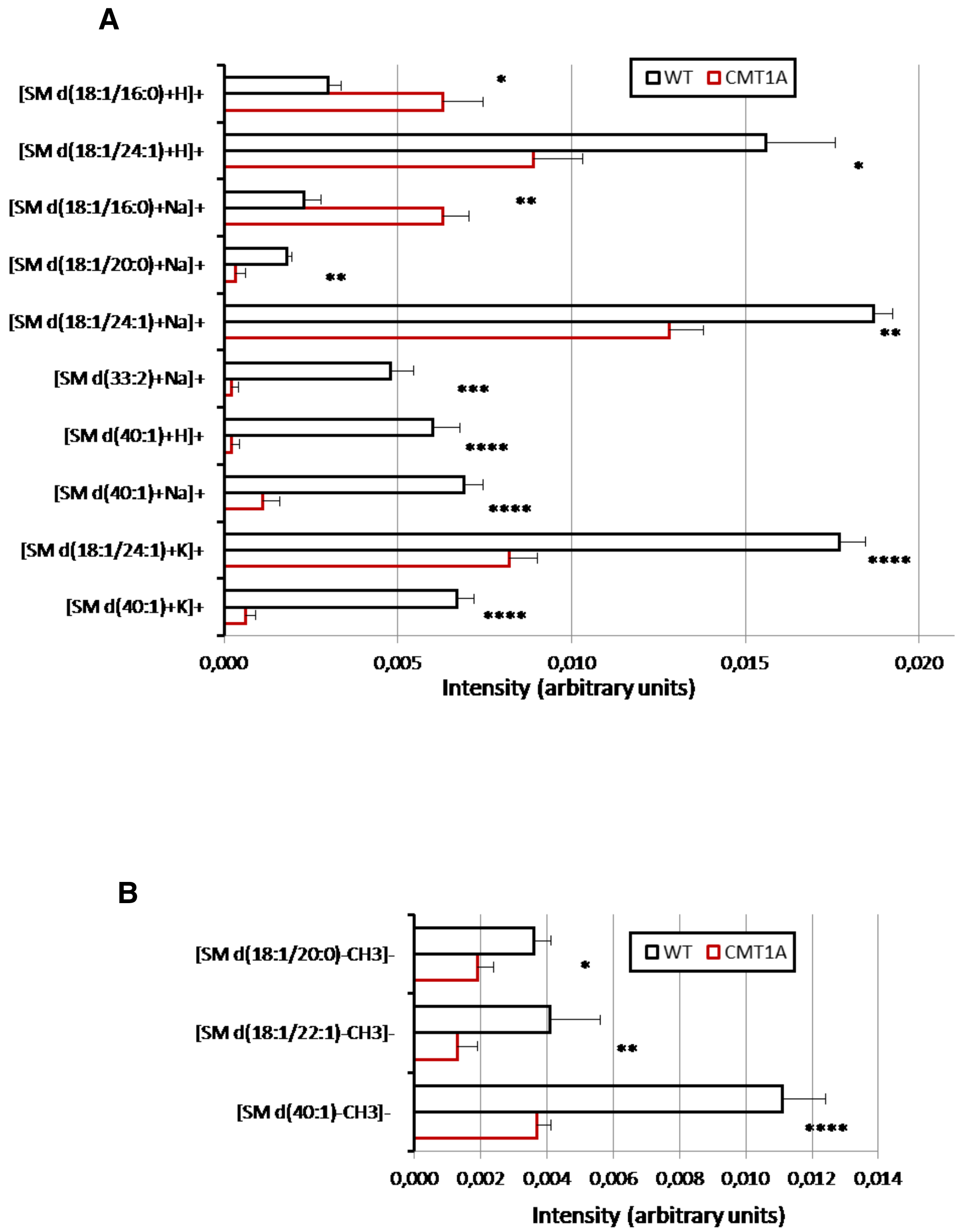

S5 Fig. MALDI-IMS on CMT1A and WT rat sciatic nerve sections.

SM detected in positive- (A) and negative-ion mode (B) that experienced a significant change in the endoneurium between WT and CMT1A samples. The species are organized in order of significance. Most of the species are more abundant in WT. Although a reduced number of species were detected in negative-ion mode, the changes observed correlated with those observed in positive-ion mode.

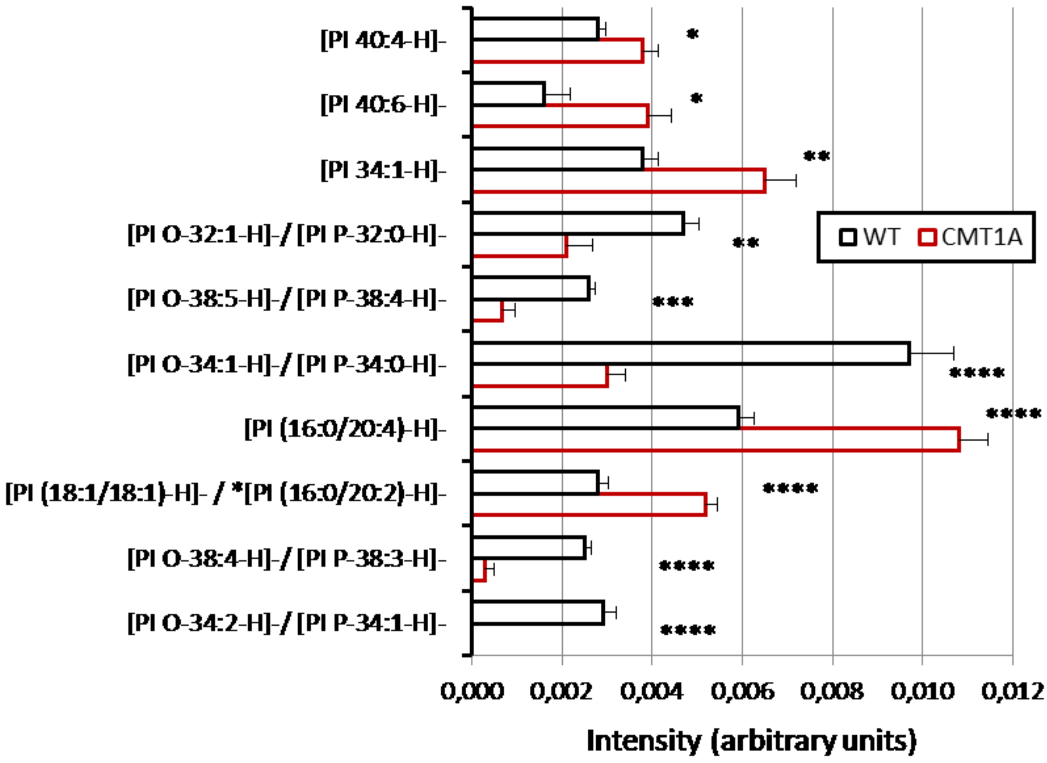

S6 Fig. MALDI-IMS on CMT1A and WT rat sciatic nerve sections.

PI and PI ether/vinyl-ether species detected in negative-ion mode in fibers that experienced a significant change between WT and CMT1A. In most cases, we could not determine if they were ether or vinyl-ether species. Diacyl species are more abundant in CMT1A and ether/vinyl ether species are more abundant in WT.

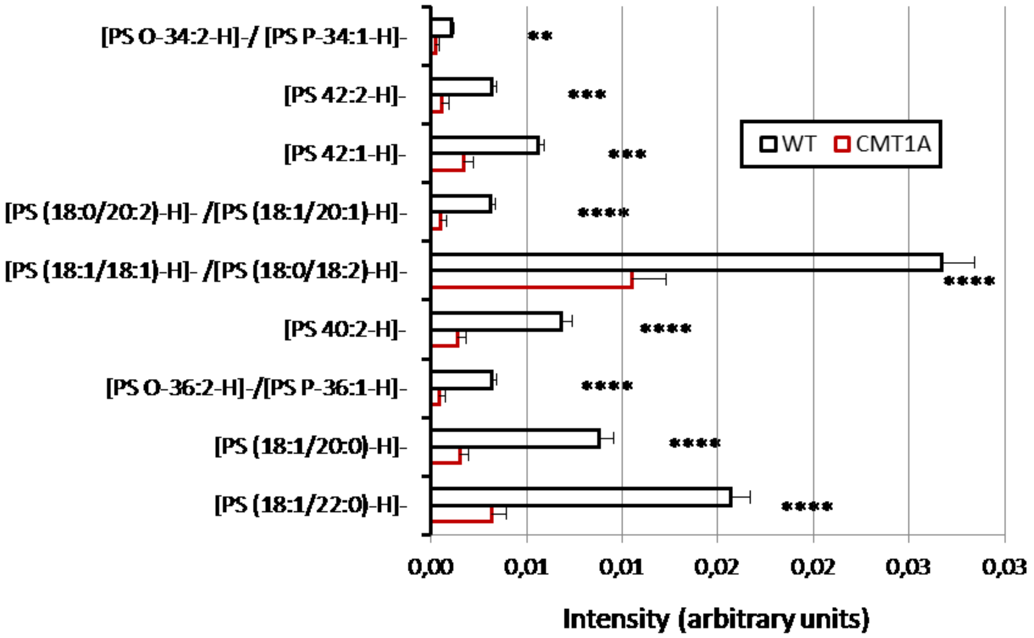

S7 Fig. MALDI-IMS on CMT1A and WT rat sciatic nerve sections.

PS and PS ether/vinyl-ether species detected in negative-ion mode in fibers that experienced a significant change between WT and CMT1A. There is a general reduction in the abundance of the species in CMT1A.

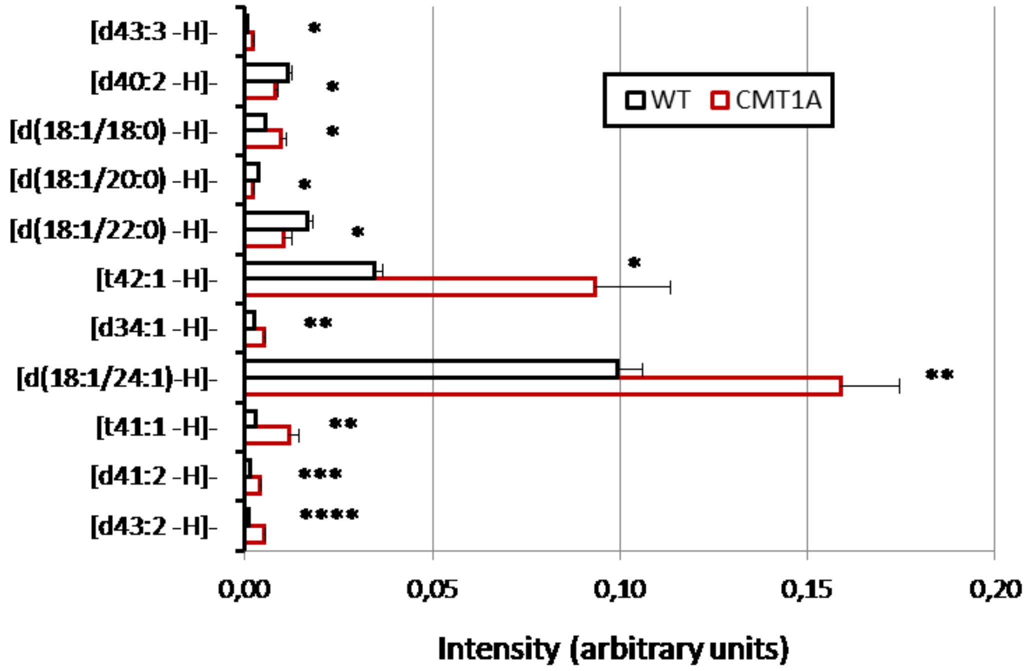

S8 Fig. MALDI-IMS on CMT1A and WT rat sciatic nerve sections.

Sulfatide species detected in negative-ion mode in fibers that experienced a significant change between WT and CMT1A. In general, there is an increase in CMT1A of the abundance of the species containing long fatty acids.

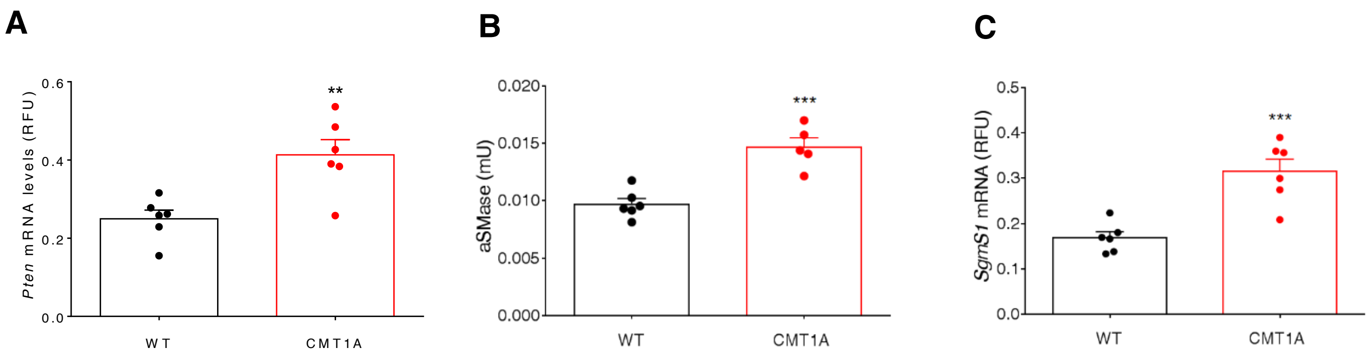

S9 Fig. Expression and activity of SP and GP pathway key enzymes are altered in CMT1A sciatic nerves.

(A) *PTEN*, a phospholipid phosphatase that inhibits myelination was significantly increased in CMT1A rat sciatic nerve (red) compared to WT littermates (black). **(B)** Also acid sphingomyelinase (aSMase) activity was increased in the transgenic nerves as well as the expression of *SMGS1*, two anabolic enzyme involved in sphingomyelin synthesis and recycling. (n = 6, unpaired t test, two-tailed). Data were represented as mean ± mean of standard error (SEM). *** = $p < 0.001$, ** = $p < 0.01$.

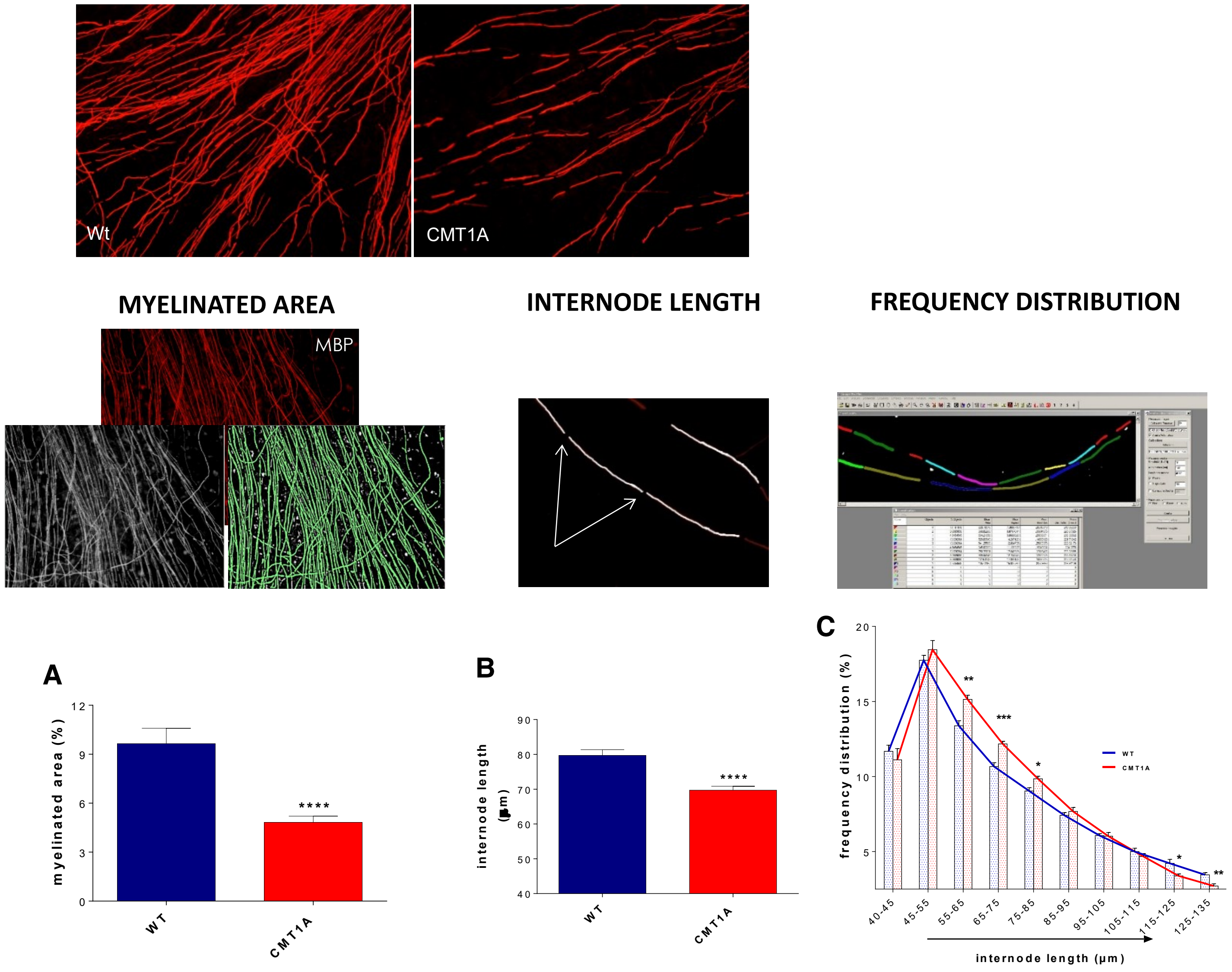

S10 Fig. Advanced quantitative neuropathology on *in vitro* myelinated fibers

We performed quantitative neuropathology on CMT1A (n=7) (red bar) and WT (n=7) (blue bar) myelinatying DRG cultures. We developed an original, semi-automated system able to accurately measure critical geometric parameters of myelinated fibers. Briefly, DRG cultures are stained with a monoclonal antibody against MBP and with the secondary goat anti-mouse ALEXA594-IgG to reveal the myelinated fibers. Then, images from the whole culture are taken (40x) with an Olympus PROVIS AX60 microscope, connected to an Olympus DP70 digital camera and stored. Finally, the images are processed by the automatic system (see also the video). Actually, this system recognizes the dysmyelinating phenotype of transgenic DRG cultures. In fact, CMT1A myelinated fibers are not merely reduced **(A)** but also display a striking shortening of internode length **(B)**. The system is also able to analyze the frequency distribution of internode length. In particular, we calculated the length of more than 10.000 internodes for each culture. Interestingly, in CMT1A DRG cultures, we demonstrated a significant enrichment of shorter internodes that is responsible for the general internode length reduction described in this and other hereditary neuropathies **(C)**. Unpaired 2-tailed t-test was used for statistical comparison in A and B. Holm-Sidak multiple comparison test after 1-way analysis of variance was used for statistical comparison in C. Data were represented as mean ± mean of standard error (SEM). **** = $p < 0.0001$, *** = $p < 0.001$, ** = $p < 0.01$, * = p < *0.05*.

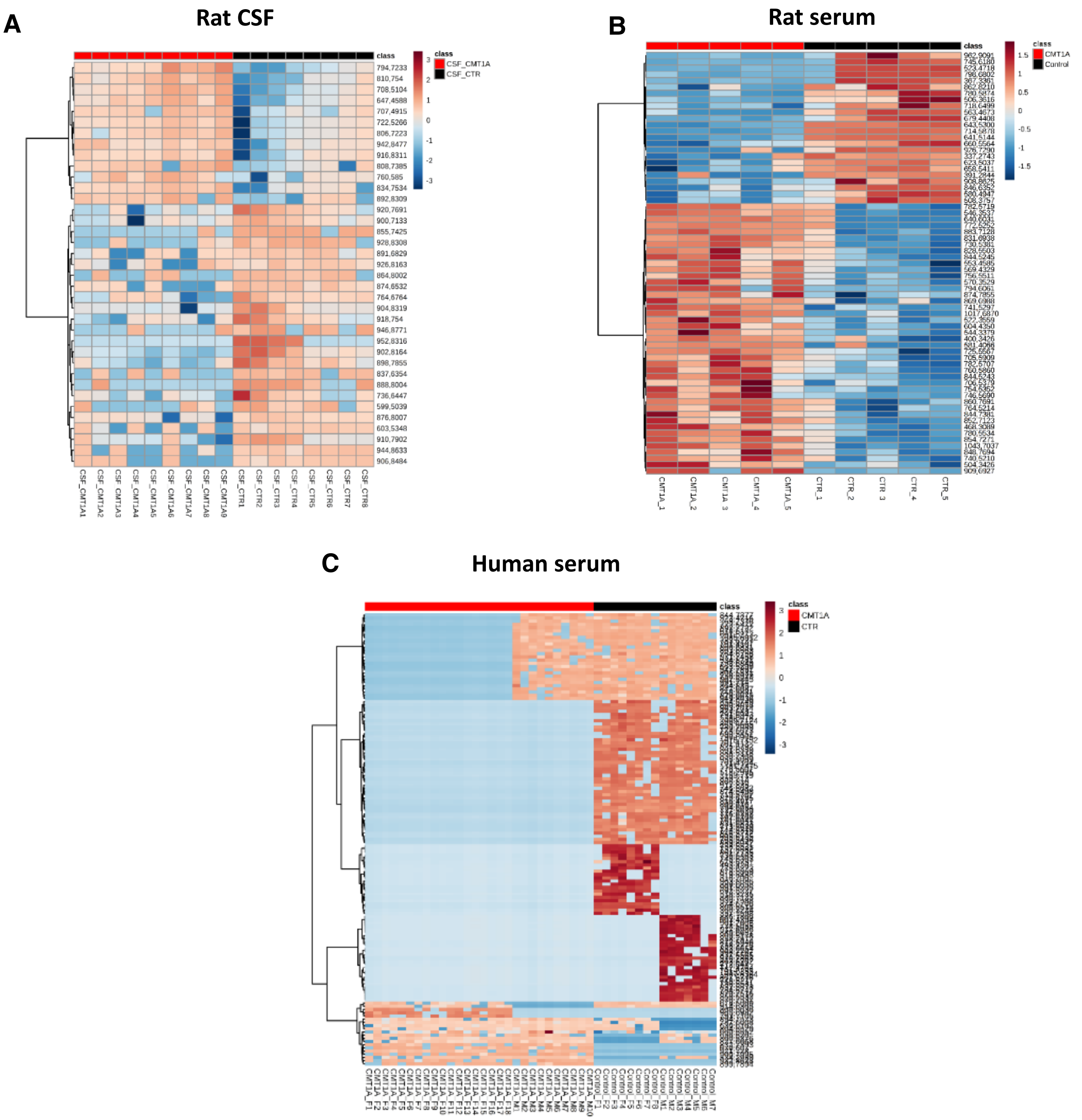

S11 Fig. Heatmaps of CSF and serum lipidome clearly discriminates CMT1A from controls. Orthogonal Projections to Latent Structures Discriminant Analysis (OPLS-DA) of untargeted lipidomics data was able to reliably discriminate CMT1A rat CSF (red, *n* = 9) from the WT one (black, *n* = 8). **(A)** Heatmap generated with the most significant features (with highest fold change and statistical significance) detected by Multivariate Data Analysis (MVA). Thirty-seven features were mainly responsible for the difference between the two groups. **(B)** Corresponding feature analysis of rat serum lipidome. We analysed WT (black, *n* = 5) and CMT1A (red, *n* = 5) profile. The two genotypes were clearly separated by OPLS-DA. Sixty-seven significant features, as shown in the relative heatmap, were responsible for the difference. **(C)** Untargeted lipidomics was also performed on the serum of 15 healthy donors and 28 CMT1A patients. One hundred and forty-one significant features, shown in the heatmap, were responsible for the observed difference.

S1 Table. Rat primers used in RT-qPCR

Gene	Primers	Amplicon size	Probe
YWHAZ	LP 5'-tcctgaactccccagagaaa-3'	108 bp	#108
NM_013011	RP 5'-gcaatggcttcatcaaaagc-3'		
HMBS	LP 5'- gcaggagttcagtgccattat-3'	82 bp	#76
NM_013168	RP 5'-cattcctctgggtgcaagat-3'		
ACTB	LP 5'-aaggccacccgtgaaaagat-3' RP	72 bp	#64
NM_031144	5'-accagaggcatacagggaca-3'		
CERS1	LP 5'-tgtgcctgacattccgtact-3'	99 bp	#63
NM 001044230	RP 5'-gctgcaaaagccacaatgta-3'		
CERS2	LP 5'-accggtcagctttgcact-3'	66 bp	#50
NM 001033700	RP 5'-cgttcccaccagaagtagtca-3'		
SGMS1	LP 5'-gggaacacacggagaagcta-3'	85 bp	#4
NM 181386	RP 5'-tggacaggtttcttccttgg-3'		
PTEN	LP 5'-cacaagaggccctggattt-3'	76 bp	#60
NM 031606	RP 5'-cgcctctgactgggaatagt-3'		
